# Blood Oxygenation Level-Dependent Response to Multiple Grip Forces in Multiple Sclerosis: Going Beyond the Main Effect of Movement in Brodmann Area 4a and 4p

**DOI:** 10.3389/fncel.2021.616028

**Published:** 2021-04-26

**Authors:** Adnan A. S. Alahmadi, Matteo Pardini, Rebecca S. Samson, Egidio D’Angelo, Karl J. Friston, Ahmed T. Toosy, Claudia A. M. Gandini Wheeler-Kingshott

**Affiliations:** ^1^Department of Diagnostic Radiology, Faculty of Applied Medical Science, King Abdulaziz University, Jeddah, Saudi Arabia; ^2^NMR Research Unit, Department of Neuroinflammation, Queen Square MS Centre, UCL Queen Square Institute of Neurology, London, United Kingdom; ^3^Department of Neuroscience, Rehabilitation, Ophthalmology, Genetics, Maternal and Child Health (DINOGMI), University of Genoa, Genoa, Italy; ^4^IRCCS Ospedale Policlinico San Martino, Genoa, Italy; ^5^Brain Connectivity Center, IRCCS Mondino Foundation, Pavia, Italy; ^6^Department of Brain and Behavioural Sciences, University of Pavia, Pavia, Italy; ^7^Wellcome Centre for Imaging Neuroscience, UCL Queen Square Institute of Neurology, University College London, London, United Kingdom

**Keywords:** BA 4p, BA 4a, fMRI, force, multiple sclerosis

## Abstract

This study highlights the importance of looking beyond the main effect of movement to study alterations in functional response in the presence of central nervous system pathologies such as multiple sclerosis (MS). Data show that MS selectively affects regional BOLD (blood oxygenation level dependent) responses to variable grip forces (GF). It is known that the anterior and posterior BA 4 areas (BA 4a and BA 4p) are anatomically and functionally distinct. It has also been shown in healthy volunteers that there are linear (first order, typical of BA 4a) and nonlinear (second to fourth order, typical of BA 4p) BOLD responses to different levels of GF applied during a dynamic motor paradigm. After modeling the BOLD response with a polynomial expansion of the applied GFs, the particular case of BA 4a and BA 4p were investigated in healthy volunteers (HV) and MS subjects. The main effect of movement (zeroth order) analysis showed that the BOLD signal is greater in MS compared with healthy volunteers within both BA 4 subregions. At higher order, BOLD-GF responses were similar in BA 4a but showed a marked alteration in BA 4p of MS subjects, with those with greatest disability showing the greatest deviations from the healthy response profile. Therefore, the different behaviors in HV and MS could only be uncovered through a polynomial analysis looking beyond the main effect of movement into the two BA 4 subregions. Future studies will investigate the source of this pathophysiology, combining the present fMRI paradigm with blood perfusion and nonlinear neuronal response analysis.

## Introduction

The primary motor cortex, M1 or Brodmann area 4 (BA 4), is very important because of its essential role in generating movement, a skill often affected by diseases such as multiple sclerosis (MS). Interestingly, BA 4 has two subregions with distinct cytoarchitectonic properties, anatomy, and neurochemistry both in humans and primates (Strick and Preston, 1982; Geyer et al., 1996; Kaas and Collins, 2002). Geyer et al. showed that the two subdivisions have differences in transmitter-binding sites and laminar density of neurons (Geyer et al., 1996). In particular, they showed that BA 4a has more densely packed pyramidal cells. On the other hand, BA 4p has higher laminar-specific densities of different receptor and transmitter binding sites.

Magnetic resonance imaging (MRI) functional studies have shown that in healthy volunteers, the blood oxygen level-dependent (BOLD) signal response is modulated by attention (Binkofski et al., 2002) and imagined forces (Sharma et al., 2008) in BA 4p, while BA 4a responds to motor control (Alahmadi et al., 2016). In other words, BA 4a is predominantly related to execution, whereas BA 4p is predominantly related to higher-order cognitive tasks (Binkofski et al., 2002; Sharma et al., 2008; Alahmadi et al., 2015b, 2016, 2017; Alahmadi, 2020).

A recent study of healthy volunteers has revealed that the BOLD response to complex motor tasks, involving different grip forces (GFs), is characterized by different MRI signal response profiles, even during observation (Keisker et al., 2009; Alahmadi et al., 2016; Casiraghi et al., 2019). Interestingly, the study reported a distinct behavior of the BOLD-GF relationship within the two subregions of BA 4 (Alahmadi et al., 2016). The BOLD-GF relationship follows a distinct nonlinear negative third-order profile within BA 4p, while it is linear in BA 4a. Moreover, in healthy volunteers, the BOLD signal within the two subregions has a distinct response to motor complexity, when using the dominant or nondominant hand while applying different GFs (Alahmadi et al., 2015b). Therefore, the functional differences between BA 4a and BA 4p provide an ideal case to compare complex BOLD responses going beyond the zero order (or main effect) of movement in a pathology like MS.

A question arises as to whether and if so how these behaviors are affected by aging and by diseases of the central nervous system, warranting a full characterization of the BOLD behavior in these two subregions, beyond a standard main effect of movement. Some investigations have reported, indirectly, that there are distinct responses in BA 4p and BA 4a in aging (Ward and Frackowiak, 2003) and in stroke patients (Ward et al., 2007). In particular, they showed that BA 4p is affected by aging and that it is key to the functional integrity of the cortical–spinal system and motor recovery in patients with stroke. Therefore, advanced analysis of BA 4a and BA 4p could highlight mechanisms of functional alterations otherwise shadowed in an undifferentiated analysis of BA 4.

On the bases of these considerations, the present study investigates the non-linear behavior of the BOLD response to different GFs within BA 4a and BA 4p, in healthy volunteers and in people with MS, a neurological disease known to affect the motor system. MS has complex disease mechanisms involving a number of pathophysiological components, including demyelination, axonal loss, and inflammation. Accumulation of sodium ions in tissue and a redistribution of sodium channels along damaged axons alters conduction properties (Paling et al., 2013; Cercignani et al., 2017), which could also be affected by alterations of tissue–blood perfusion (Rovaris et al., 2002; Rashid et al., 2004; Rocca et al., 2007; Paling et al., 2014; Bester et al., 2015; Lapointe et al., 2018). fMRI studies in MS have shown altered patterns of activations (Rocca et al., 2007; White et al., 2009; Filippi et al., 2013) and altered resting state networks (Castellazzi et al., 2018), but were not designed to answer questions about complex BOLD behavior.

The hypothesis of this study is therefore, in MS, there is (1) an altered functional response in BA 4 compared with healthy volunteers during a motor fMRI task and that (2) this alteration is region specific. Given the involvement of BA 4p in higher-order motor control and its modulation by attention and task complexity, we also hypothesized that (3) area BA 4p may show more severe abnormalities than BA 4a in the presence of MS pathology when compared with healthy volunteers. If this is true, then the BOLD-GF relationship in BA 4a and BA 4p may show different regional patterns of alteration compared with healthy volunteers, offering new insights in the pathology of MS.

## Materials and Methods

### Subjects

Fourteen right-handed healthy volunteers [nine females, five males; mean age 31(±4.64) years] and 14 right-handed relapsing remitting MS (RRMS) patients [10 females, four males; mean age 35(±5.36) years; median (range) expanded disability status score (EDSS) 3.5 (1.5–6.5)]; median (range) 9-Hole Peg Test (9-HPT) = 20.05 (14.7–33.1) were recruited. The handedness of subjects were assessed according to the Edinburgh handedness scaling questionnaire (Oldfield, 1971). All subjects gave informed consent, and the study was approved by the local research and ethics committee.

### Magnetic Resonance Imaging Acquisition

A 3.0 T MRI scanner, Philips Achieva system (Philips Healthcare, Best, Netherlands), and a 32-channel head coil were used. The imaging acquisition protocol included the following: T1-weighted volume (3DT1): 3D inversion-recovery prepared gradient-echo (fast field echo) sequence with inversion time (TI) = 824 ms, echo time (TE)/repetition time (TR) = 3.1/6.9 ms, flip angle = 8° and voxel size = 1 mm isotropic; BOLD sensitive T2^∗^-weighted echo planar imaging (EPI): TE/TR = 35/2,500 ms, voxel size = 3 × 3 × 2.7 mm^3^, inter-slice gap of 0.3 mm, SENSE factor = 2, number of slices = 46, acquired with descending order, field of view = 192 × 192 mm^2^, number of volumes = 200, number of dummy scans = 5, flip angle = 90°.

### fMRI Paradigm

The experimental design was a *visually* guided event-related fMRI paradigm, where subjects used their right (dominant) hand to squeeze a rubber ball with varying GF levels. The design comprised five GF targets [20, 30, 40, 50, and 60% of subjects’ maximum voluntary contraction (MVC)] interleaved with rest intervals, each repeated randomly 15 times. This paradigm has been validated previously in studies of healthy volunteers (Alahmadi et al., 2015b, 2016, 2017; Casiraghi et al., 2019).

Before the fMRI session, subjects were given a 6 min training session including learning, watching, and performing a similar but not identical paradigm. During the fMRI session, participants lay supine on the scanner bed throughout the experiment and were instructed to extend both of their arms in a relaxed comfortable position. A support hand pad was provided for each subject to ensure comfort and compliance.

### Image Pre-processing and Analyses

Data processing was performed using statistical parametric mapping SPM12^1^ implemented in Matlab14b (Mathworks, Sheborn, MA). The pre-processing steps for each subject adopted the following fMRI pipeline: (i) slice time corrections, (ii) spatial volume realignments, (iii) co-registration with the 3DT1 volume, (iv) normalization with the tissue probability maps of SPM12, and (v) smoothing of the functional volumes with an 8 mm isotropic full-width half maximum (FWHM) Gaussian kernel.

### Statistical Analysis

#### Within-Subjects (First-Level Analysis)

Signal changes were modeled using a polynomial expansion as described in Alahmadi et al. (2016) and according to Buchel et al. (1996, 1998). Briefly, for each subject, a fixed effect analysis was performed. To test efficiently for linear and nonlinear correlations between BOLD signal response and the applied grip force recorded throughout the experiment, a parametric design was chosen. The parametric design included a set of orthogonalized polynomial orders (up to the fourth order) and was specified by the integral of the forces for each subject. The rationale for choosing four polynomial orders was based on the specific design of our study, i.e., the number of forces applied (five different levels). This step creates five regressors of interest: zeroth order represents the main effect of gripping regardless of the applied force (i.e., simply detecting regions that activate in response to gripping irrespective of any correlation between gripping force and BOLD signal), first order represents any linear associations between BOLD signals and the applied forces (i.e., detecting regions where if the force increases, there is a linear increase in BOLD activity), and second to fourth orders represent nonlinear effects modeling the relationships between BOLD signals and the applied forces. For example, the positive second-order effect represents a U-shaped profile where the BOLD signal is decreased for middle-range applied forces and increased at lower and higher applied forces. These covariates were then convolved with a canonical hemodynamic response function (HRF) for standard SPM and general linear model (GLM) analysis (Friston et al., 1995). The subject’s movement-related realignment parameters were included as regressors of no interest in each GLM (Friston et al., 1996). At this within-subject level, *t*-statistics was used to test for the effects of the polynomial coefficients.

#### Between-Subjects (Second-Level Analysis)

Contrast images from the within-subject analysis for the five polynomial orders were entered into random effect analyses, testing for nonlinear effects within and between groups, with the appropriate *t*-tests (i.e., one-sample *t*-test for the within-group tests and two-sample *t*-test for between group comparisons). Significant voxels were defined using *P*< 0.05, corrected for multiple comparisons (FWE). Importantly, the number of comparisons (voxels) performed in this study was within the subregions of BA 4; thus, the numbers of comparisons were low compared with the whole brain.

**FIGURE 1:**
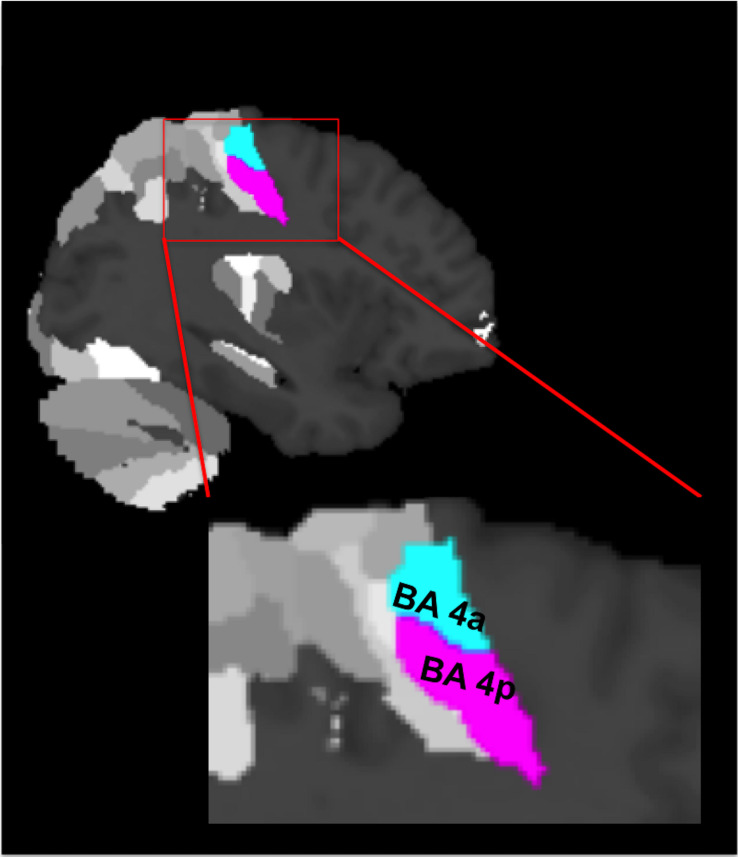
The cytoarchitectonic assignments of BA 4a and BA 4p projected onto the maximum probability map of the brain as provided by the SPM anatomy toolbox.

In addition to the above whole brain SPM analysis, BA 4 was defined and subdivided according to Eickhoff et al. (2005) as guided by Geyer et al. (1996; Figure 1). The study by Eickhoff et al. (2005) introduced a new probabilistic cytoarchitectonic map of different brain regions (including BA 4), which significantly improved the accuracy of anatomical labeling. The new cytoarchitectonic map measured the probability of a single voxel falling within an area based on 10 post-mortem brains normalized to the MNI single-subject template. Only voxels (or clusters) having the highest probability of falling within the MNI subdivision of BA 4 were considered for each *post mortem* subject and were used to define a map of BA 4 in MNI space. Since this is an *a priori* predefined region of interest (ROI), we used this anatomical ROI for each subject and performed small volume correction (SVC) to increase our sensitivity when looking at individual subject behaviors and parametric effects. To assess the relationship between BOLD signal and GF, we plotted the average group signal estimate over voxels within these predefined ROIs for either BA 4a or BA 4p. Each plot therefore reports the maximum likelihood estimates of the mapping between the different applied GF and BOLD signals based on the polynomial expansion (Alahmadi et al., 2016).

To better understand the effect of disability on BOLD responses to GF, we divided the MS group based on their EDSS score into two subgroups of low (EDSS ≤ 3) and high disability (EDSS > 3). The 9-HPT was highly correlated with the EDSS scores and thus the two groups could be subdivided similarly to low (9-HPT ≤ 21.7) and high disability (9-HPT > 21.7). Following the subdivision of the MS group, the low EDSS group comprised eight subjects [four females, four males; mean age 33.8(±4.5) years; median (range) EDSS 1.5 (1.5–2.5); median (range) 9-Hole Peg Test (9-HPT) = 19.1 (14.7–22.1)], while the high EDSS group comprised the remaining six subjects [five females, one male; mean age 36.1(±7.8) years; median (range) EDSS 6 (3.5–6.5); median (range) 9-Hole Peg Test (9-HPT) = 25 (22.3–33.1)].

In order to characterize the response profile of each subdivision in each group, we used the relative contribution of each polynomial term as described in Alahmadi et al. (2015b). We therefore classified the effect of GF at each voxel within BA 4a and BA 4p based on the polynomial order that showed the highest standardized group effect size (i.e., the most significant group difference).

## Results

All subjects were able to perform the task correctly (Table 1).

**TABLE 1:** Grip force task performance, showing the average (±SD) maximum voluntary contraction (MVC) (%) and duration (s) of squeeze for healthy and multiple scelerosis (MS) subjects.

	**20%**	**30%**	**40%**	**50%**	**60%**
**(A) Healthy volunteers**					
**MVC %**	21.23 ± 2.28	30.23 ± 1.68	39.26 ± 2.29	51.23 ± 1.28	59.11 ± 1.32
**Duration (s)**	2.89 ± 0.34	3.19 ± 0.33	3.11 ± 0.14	2.99 ± 0.51	3.13 ± 0.22
**(B) MS**					
**MVC %**	22.32 ± 1.89	31.32 ± 1.34	40.17 ± 2.15	51.32 ± 1.49	60.39 ± 1.29
**Duration (s)**	3.04 ± 0.75	2.97 ± 0.95	3.25 ± 0.093	3.08 ± 0.693	3.32 ± 1.12

In this study, we report five major findings:

### Main Effect of Movement

**FIGURE 2:**
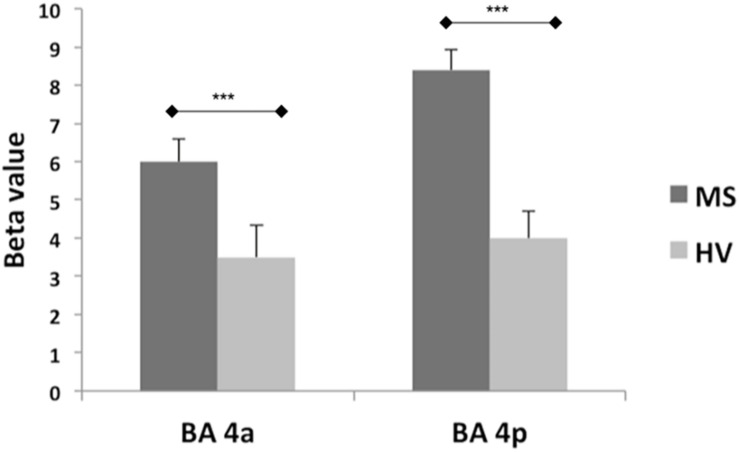
Mean of the beta values and their standard errors calculated at group level for the main effect of gripping for both groups and sub-regions. There are significantly higher betas (***p = 0.001) in the MS compared to the Healthy volunteers within both sub-regions (BA 4a and 4p).

**FIGURE 3:**
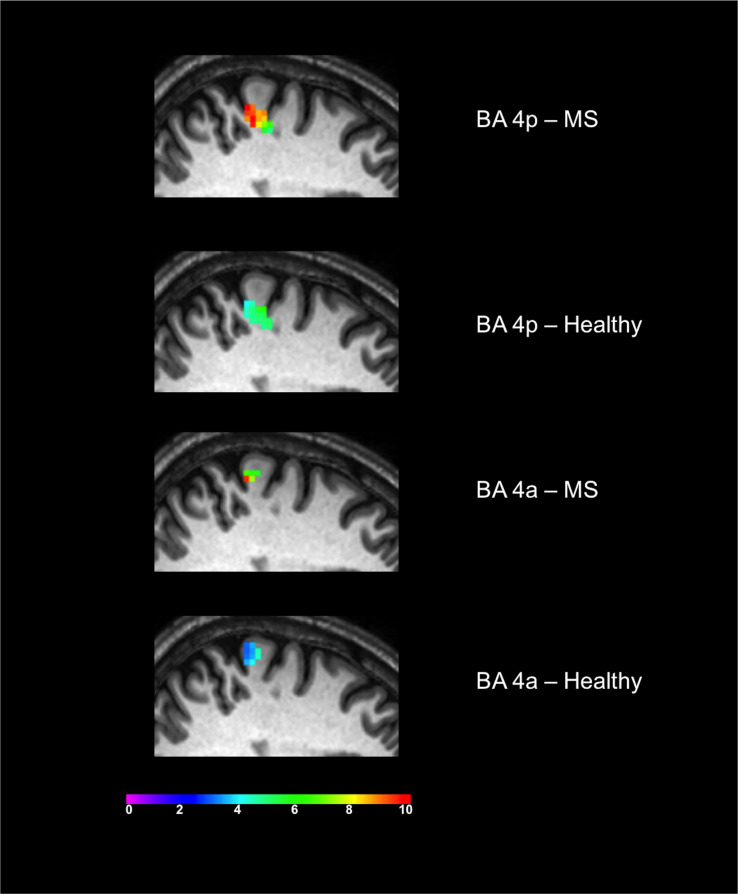
Significant activations of the main effect of gripping within BA 4p and BA 4a in both MS and Healthy volunteers. Colors represents the *T*-value of the effects at a 0.05 FWE.

**FIGURE 4:**
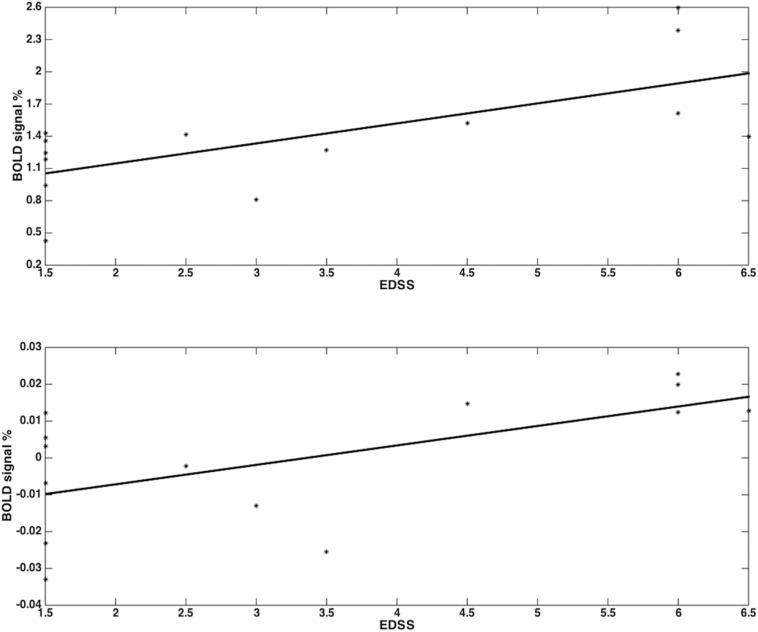
RRMS patients showed increased activations as their EDSS increased within BA 4p (*p* = 0.001; *r* = 0.68) (top plot) and within BA 4a (no sgificant results) (bottom plot) in the main effect of gripping (i.e., 0th order).

Both groups activated BA 4a and BA 4p (Figures 2, 3). RRMS patients showed increased and greater activation extent compared with healthy volunteers in both BA 4a and BA 4p subregions (Figures 2, 3) (*p* = 0.001). RRMS patients also showed increased activations as their EDSS increased within BA 4p only (*p* = 0.001) (Figure 4).

### Average Blood Oxygen Level-Dependent Relationship With Grip Forces in Brodmann Area 4a

**FIGURE 5:**
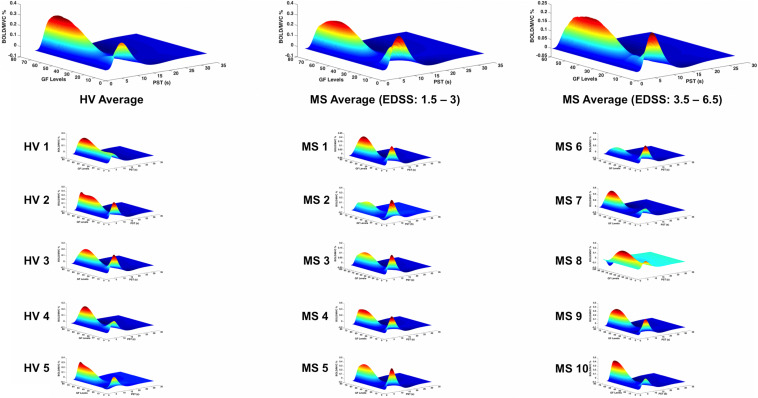
BOLD responses (*Z*-axis) of the fitted polynomial-orders of GF (*Y*-axis) at the defined post-stimulus time (PST) (*X*-axis) within BA 4a for Healthy volunteers (HV 1-5), MS patients with low (MS 1-5) and high EDSS (MS 6-10)—representing an estimate of the mapping between GF and BOLD based on all components of the polynomial expansion. The top row shows the average group effect while underneath examples of individual subjects are plotted.

In both groups, significant relationships were detected between BOLD signal and GF. There were no differences detected between MS subjects and healthy volunteers in terms of the relationship between BOLD signal and GF within BA 4a (Figure 5).

### Average Blood Oxygen Level-Dependent Relationship With Grip Forces in Brodmann Area 4p

**FIGURE 6:**
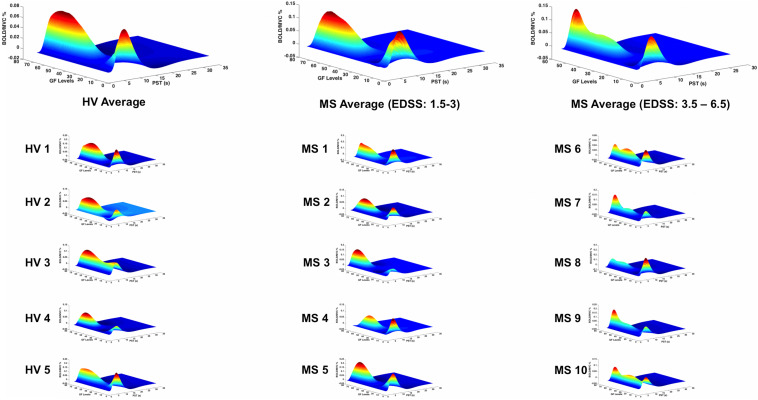
BOLD responses (*Z*-axis) of the fitted polynomial-orders of GF (*Y*-axis) at the defined post-stimulus time (PST) (*X*-axis) within BA 4p for Healthy volunteers (HV 1-5), MS patients with low (MS 1-5) and high EDSS (MS 6-10)—representing an estimate of the mapping between GF and BOLD based on all components of the polynomial expansion. The top row shows the average group effect while underneath examples of individual subjects are plotted.

In patients with low EDSS, the BOLD–GF relationship was very similar to healthy volunteers (mainly negative third order), whereas at higher EDSS, the predicted BOLD versus GF deviated from the healthy volunteers’ pattern and the BA 4a pattern (Figure 6, third column).

### Grip Force Response Profile in Single Subjects

Interestingly, the profile of the mean BOLD signal versus GF was very similar across individual subjects, when grouping subjects by disease stage. Figures 5, 6 show the maximum likelihood estimates of the mapping between the applied GF and BOLD signals based on the polynomial expansion at the group and subject levels.

### Categorizing Effect Sizes

By performing a *post hoc* analysis to identify the polynomial order showing the highest effect size, we showed that in both groups (healthy and MS) the first-order effect was predominant within BA 4a. On the other hand, the predominant effect within BA 4p was different in the two groups. In healthy volunteers, a negative third-order effect was predominant, while in MS, a positive (U-shaped) second-order effect was predominant, with BOLD signal increasing with the highest force in MS, which elicited the lowest BOLD response in healthy volunteers (see Supplementary Material).

## Discussion

In this study, we demonstrate the importance of characterizing high-order BOLD responses to GF as opposed to simply assessing group differences in the main effect of movement (zero order) between patients and healthy controls in pathologies such as MS. This characterization was performed within the subdivisions of the primary motor region (BA 4a and BA 4p), which present a different order response to the intensity of movement (Alahmadi et al., 2015a, 2016; Alahmadi, 2020). The main findings are that there are distinct grip (i.e., main effect) and force level effects within these two subregions in healthy and MS subjects. We further demonstrated that the main effect of gripping and the non-linear BOLD–GF relationship within BA 4p change with disease progression.

A key finding is that the non-linear relationship between BOLD response and GF was confirmed in both healthy volunteers and MS groups and that in people with MS, it was distinctly altered only in BA 4p (Figure 6). This result can be supported by the fact that the neuronal and cellular structures as well as chemistry of these two subregions are different (Strick and Preston, 1982; Geyer et al., 1996; Kaas and Collins, 2002). Thus, pathologies such as MS could additionally affect these two subregions differentially. Focusing on BA 4a and 4p enabled us to characterize the BOLD signal response based on its relationship to increasing GF, assessing its sensitivity to pathological changes at a subregional level.

BA 4 as a whole responded to the main effect of grip in both MS and healthy control groups. This is in line with previous motor gripping studies that showed the role of BA 4 in motor generation and function (Kuhtz-Buschbeck et al., 2001; Rocca et al., 2007; Kuhtz-Buschbeck et al., 2008; Keisker et al., 2009; White et al., 2009).

This distinct functional segregation and parametric responses was effectively captured by this study. Indeed, our findings regarding the main effect of movement show that both subregions are activated within both groups. The responses though are different within the two subregions as the BOLD signal was significantly increased in MS compared with the healthy group, especially in BA 4p (Figures 2, 3). Previous reports of motor functional studies illustrate different outcomes in the main effect of movement (within BA 4 as a whole) in MS, with the BOLD signal response shown to either increase (Lee et al., 2000; Reddy et al., 2000; White et al., 2009) or to have no difference compared with healthy volunteers (Mancini et al., 2009; White et al., 2009). Unfortunately, most of these studies used automated anatomical labeling that relies on a template (e.g., the Talairach) or manual eye assessment labeling, both of which are highly prone to inaccuracy and difficult to generalize. The other limiting factor is that most of these studies were not anatomically specific for BA 4a or BA 4p [e.g., both M1 and S1 (primary sensory area) were labeled as “sensorimotor cortex” or SMC]. This makes comparisons between our results and earlier studies difficult. The inconsistency among earlier studies can be attributed to factors such as differences in paradigms, number of subjects, threshold values, and MS subtypes. The last factor, i.e., MS subtype, may be very significant in affecting outcome as functional activations have been shown to be altered during disease progression (Rocca et al., 2005). More importantly, it has been suggested that MS patients during the early stage of the disease tend to have normal patterns of activation (Pantano et al., 2015).

This study goes beyond the main effect of movement and shows that the non-linear relationship BOLD–GFs within BA 4 is complex and region specific. In previous studies of healthy volunteers, we have demonstrated the complexity of BOLD signals as a function of GF in different motor, submotor, associative, and cerebellar areas (Alahmadi et al., 2015b, 2016, 2017; Casiraghi et al., 2019). These were seen as well in action observation and execution networks (Casiraghi et al., 2019). In the present study, we show that pathology affects the BOLD–GF relationship differently in BA 4a and BA 4p and that the altered behavior was indicative of high EDSS. What was particularly striking was the consistency of the profile between individual subjects, reflecting the group level findings (Figures 5, 6). It is not possible from the present data to interpret the origin of the different GF–BOLD relationships, which need further investigation with multimodal studies and possibly cellular physiology experiments. Our data show that complex changes in the relationship between the GF and BOLD signal are more evident within BA 4p in subjects with MS compared with healthy controls; given that previous studies confirm a more complex physiology of this subregion (Binkofski et al., 2002; Sharma et al., 2008), involved not only in force control but also in higher order functions such as attention, it is possible that a combination of these physiological processes is the source of such altered BOLD–GF dependencies. In addition, in healthy subjects and within BA 4a sub-region, the findings suggest that there is a very small BOLD signal response to the lower GF of 20%, in agreement with a linear positive correlation between the strength of GF and the BOLD signal. This behavior was, however, altered in MS subjects especially within BA 4p, which could suggest a dysfunction affecting functional response in this subregion.

The observation that the BOLD response to different GFs within BA 4p was similar to that of healthy volunteers in patients with low EDSS, while it was consistently altered at higher EDSS poses interesting mechanistic questions, suggesting that differences not only in cytoarchitecture but also in chemoarchitecture and myeloarchitecture of these two subregions may translate into differences in their susceptibility to MS pathology. For example, these architecture properties as well as differences in the distribution of neural cell densities within the two subregions (Geyer et al., 1996) could be altered or be the cause of alterations in myelination, axonal loss, vascular or neuronal activity in MS. One could speculate that given the rich density of neurotransmitters in BA 4p compared with BA 4a (Geyer et al., 1996), our observations could reflect an impaired neuronal response. With the present data, though, it is not possible to link the present functional findings to tissue microstructure alterations, nor to infer a causal relationship between an impaired functional response to a complex task and blood perfusion, microvascular response, or even sodium channel malfunction, all of which are known to be regionally affected and potentially responsible for our observations. Furthermore, these differences could be due to differences in their structural connectivity to other brain regions that could potentially drive this different behavior. Thus, our findings underline the need for multi-modal and longitudinal studies that could include other quantitative techniques such as diffusion weighted imaging (DWI) (to assess tissue microstructure and connectivity), MR spectroscopy (to assess metabolic changes), sodium imaging (to assess effects of sodium ions tissue distribution essential for neurotransmission), gray matter-sensitive sequences (to assess gray matter atrophy and lesions), and perfusion imaging to pin down mechanistic hypothesis and deliver sensitive and specific *in vivo* imaging biomarkers of the functional substrate of MS alterations; we believe that it is really important to go beyond reporting the main effect of movement, i.e., a change in BOLD signal amplitude.

The fact that these behaviors reflected EDSS association is also of interest. Previous studies have suggested that changes in functional activations in MS are possibly related to compensatory mechanisms (Staffen et al., 2002; Audoin et al., 2003; Mainero et al., 2004b; Lenzi et al., 2007), which could also be advocated to explain the higher activations observed in our data when considering the main effect of gripping in MS compared with healthy volunteers. It should be noted, however, that previous studies showed that BA 4p is involved in executive motor function tasks compared with BA 4a. The predominant reported factors for BA 4p involvement were attention (Binkofski et al., 2002), complexity (Alahmadi et al., 2016), and imagination (Sharma et al., 2008). In the current study, these factors, especially attention and complexity, are all invoked by task execution. The use of an increased GF increases the complexity of performance and the use of visual feedback to reach (as quickly as possible) and maintain (as precisely as possible) forces at a specific level (especially low and high GFs) requires increased attention. Previous reports showing that in MS there are functional alterations with task complexity (Filippi et al., 2002) and attention (Mainero et al., 2004a) may support our finding of increased functional changes in BA 4p. Given that our data shows a correlation between increased BOLD in BA4p and EDSS (an indicator of disability), we challenge the interpretation of compensation usually associated with increased BOLD response. Indeed in Castellazzi et al. (2018), it was suggested that BOLD signal increase that correlates with a worsening of the pathology may be considered a negative, hence maladaptive, mechanism.

Advanced network modeling of the functional signal behavior may assist in understanding the source of these alterations (Friston et al., 2003). Dynamic causal modeling (DCM) would be a possible analysis to perform to help understand the precise nature of the non-linearity in the BOLD response at the neuronal or hemodynamic level. Given that the most interesting hemodynamic nonlinearities emerge over a timescale of seconds, due to hemodynamic saturation effects (Friston et al., 1996, 1998, 2000, 2003), a revised paradigm that includes variations in the GF duration as well as strength would be desirable for future experiments. These findings could be also helpful in differentiating stages of MS as well as follow-up treatment effects and recovery rates, given that they are consistent at subject level. However, further analysis and data are needed in order to assess possible use in clinical routine or clinical trials. The information obtained from studying the BOLD–GF relationship could reflect disease activity, which could be helpful in developing treatment plans and monitoring recovery.

## Limitations and Methodological Considerations

In this study, there are some methodological considerations to disclose. The number of subjects could be considered to be relatively low. This is especially relevant when subdividing the MS subjects into two different groups; however, the basis of the subdivision of the groups was well defined and explained, as well as the fact that this was an exploratory study investigating only one subtype of MS. Given the striking results, though, it is important to report these findings, which could drive larger prospective studies. Future studies should also consider investigating how these results change with MS subtypes, including clinically isolated syndromes as well as progressive patients. Another limitation of this study is the inability to investigate lesions within the targeted ROIs (i.e., BA 4). Gray matter lesions in MS have been reported using MRI (Geurts et al., 2011; Sethi et al., 2012). However, gray matter lesion sequences were not planned here, although the authors advocate the need to include a gray matter lesion sequence in future functional MRI studies of MS.

## Conclusion

BA 4 has two subdivisions (BA 4p and BA 4a) that are anatomically and functionally distinct; therefore, BA 4 lends itself very well to the investigation of subregional differences in functional response to complex motor tasks in healthy and MS subjects. Here, we demonstrated region-specific alterations in the BOLD response to movement, showing that we should investigate beyond the main effect to unveil altered nonlinear coupling between the BOLD signals and GFs, especially within BA 4p. The information obtained from studying the BOLD–GF relationship could reflect not only disease activity but also the degree of morbidity that could be helpful in following treatments and recovery. Furthermore, the alteration of the BOLD–GF profile in high EDSS patients compared with healthy volunteers is a very interesting finding that opens an entire new set of avenues to study the mechanisms of neurological diseases *in vivo*. For example, the consistent alteration of the BOLD–GF profile in BA 4p in the advanced stage of MS could be explained by many factors: perhaps demyelination of BA 4p itself, which could make it unable to support an efficient functional response, or perhaps white matter fibers subtending BA 4p have redistributed sodium channels that—instead of supporting functionality—impair efficient neurotransmission, or perhaps in MS, the microvascular response is impaired with a regional specificity that makes BA 4p responding differently than BA 4a. These questions highlight the need for multi-modal cross-sectional and longitudinal studies that aim at disentangling the contribution to functional alterations of many factors, in order to pin down sensitive and specific *in vivo* imaging biomarkers.

## Data Availability Statement

The data supporting the conclusions of this article will be made available by the authors upon request, without undue reservation.

## Ethics Statement

The studies involving human participants were reviewed and approved by the Joint UCL/UCLH Committees on the Ethics of Human Research. The patients/participants provided their written informed consent to participate in this study.

## Author Contributions

AA, ATT, and CGWK conceptualized the study. AA, KJF, and CGWK designed and performed the analyses. All authors provided support, guidance with data analysis and interpretation, contributed to the article, and approved the submitted version.

## Conflict of Interest

The authors declare that the research was conducted in the absence of any commercial or financial relationships that could be construed as a potential conflict of interest.

## Footnotes

1 www.fil.ion.ucl.ac.uk/spm
