# Supplementary figures and images for: Blood Oxygenation Level-Dependent Response to Multiple Grip Forces in Multiple Sclerosis: Going Beyond the Main Effect of Movement in Brodmann Area 4a and 4p
Source: Front Cell Neurosci. 2021 Apr 26;15:616028. doi: 10.3389/fncel.2021.616028 (PMC8109244; doi:10.3389/fncel.2021.616028)

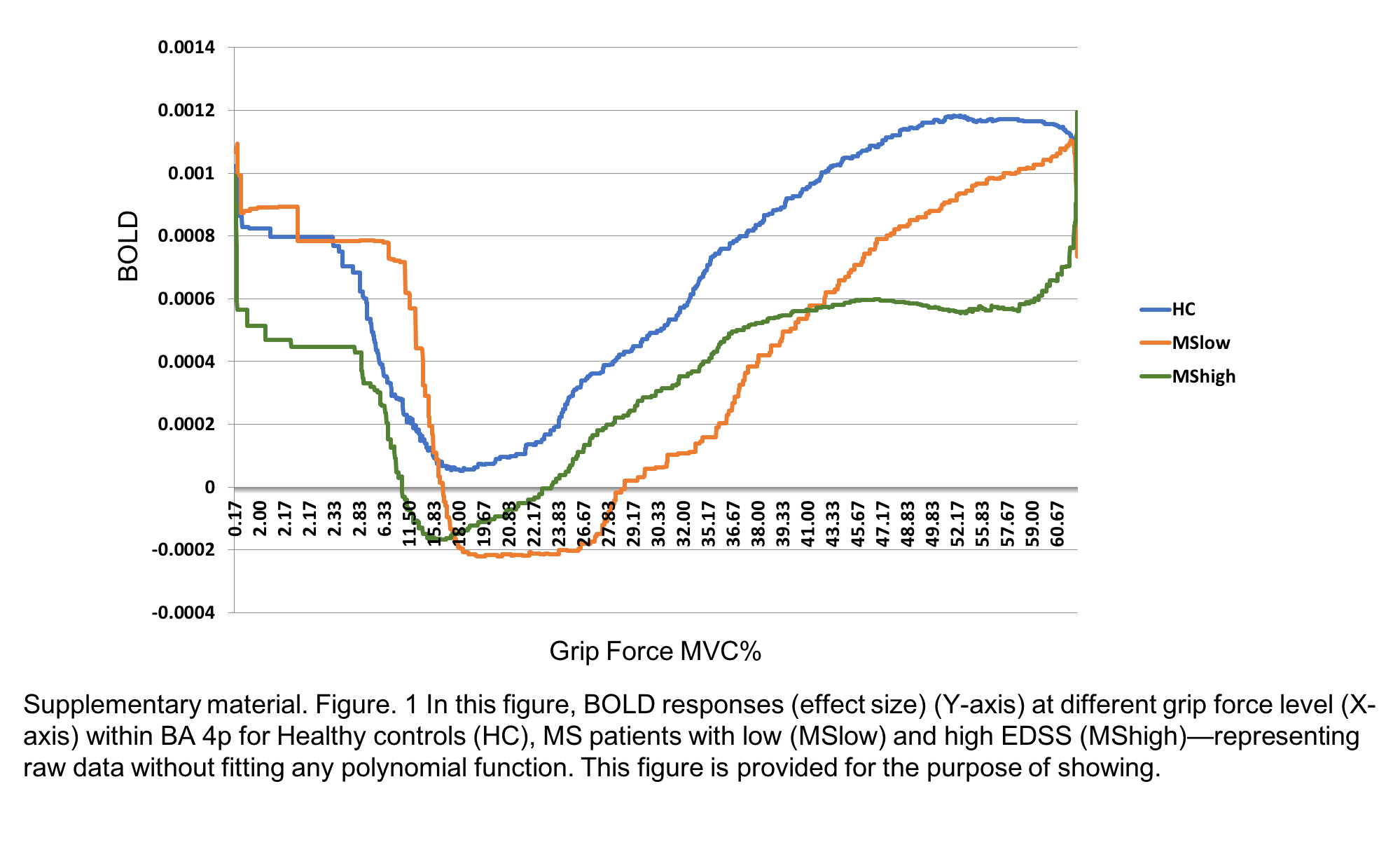

Supplement: Supplementary file 1 [file Image_1.PNG]
